# Maternal 75-g OGTT glucose levels as predictive factors for large-for-gestational age newborns in women with gestational diabetes mellitus

**DOI:** 10.1590/2359-3997000000126

**Published:** 2016-01-01

**Authors:** Krstevska Brankica, Velkoska Nakova Valentina, Simeonova Krstevska Slagjana, Jovanovska Mishevska Sasha

**Affiliations:** 1 Endocrinology Diabetes and Metabolic Disorders Clinic Medical Faculty Skopje R. Macedonia Endocrinology Diabetes and Metabolic Disorders Clinic, Medical Faculty, Skopje, R. Macedonia; 2 Faculty of Medical Science Goce Delcev University Stip R. Macedonia Faculty of Medical Science, Goce Delcev University, Stip, R. Macedonia; 3 Gynecology and Obstetric Clinic Medical Faculty Skopje R. Macedonia Gynecology and Obstetric Clinic, Medical Faculty, Skopje, R. Macedonia

**Keywords:** Gestational diabetes, oral glucose tolerance test (OGTT), large for gestational age

## Abstract

**Objective:**

Our goal was to investigate which glucose measurement from the 75-g oral glucose tolerance test (OGTT) has more capability of predicting large for-gestational-age (LGA) newborns of mothers with gestational diabetes mellitus (GDM).

**Subjects and methods:**

The study group consisted of 118 consecutively pregnant women with singleton pregnancy, patients of Outpatients Department of the Endocrinology, Diabetes, and Metabolic Disorders Clinic. All were prospectively screened for GDM between 24^th^ and 28^th^ week of pregnancy and followed to delivery. Outcome measures included: patients’ ages, pre-pregnancy BMI, BMI before delivery, FPG, 1 and 2 hour OGTT glucose values, haemoglobin A1c at third trimester, gestational week of delivery, mode of delivery and baby birth weight.

**Results:**

From 118 pregnancies, 78 (66.1%) women were with GDM, and 40 (33.9%) without GDM. There were statistically significant differences (30.7 versus 5.0%, p < 0.01) between LGA newborns from GDM and control group, respectively. Gestation week of delivery and fasting glucose levels were independent predictors for LGA (Beta = 0.58 and Beta = 0.37 respectively, p < 0.01). Areas under the receiver operator characteristic curve (AUC) were compared for the prediction of LGA (0.782 (0.685-0.861) for fasting, 0.719 (0.607-0.815) for 1-hour and 0.51 (0.392-0.626) for 2-hour OGTT plasma glucose levels).

**Conclusion:**

Fasting and 1-hour plasma glucose levels from OGTT may predict LGA babies in GDM pregnancies.

## INTRODUCTION

Gestational diabetes mellitus (GDM) is a common medical condition defined as a carbohydrate intolerance that begins or is first diagnosed during pregnancy (1). Maternal supply of carbohydrates leads to fetal hyperglycaemia, which in turn stimulates fetal pancreatic islet cells and causes hyperinsulinaemia (2). Stimulation of the insulin-sensitive tissue results in increased fetal growth, predominantly of the abdomen, and delivery of large for gestational-age (LGA) newborns (3). Both women with large fetuses and LGA newborns are at a higher risk of complications (4).

Oral glucose tolerance test (OGTT) is accepted as a diagnostic “gold standard” for GDM diagnosis. The National Diabetes Data Group (NDDG) first introduced the 3-h 100-g OGTT test as diagnostic test for GDM. This test has ability to predict postpartum diabetes mellitus in women with GDM. Later, the WHO adopted the 2-h 75-g OGTT recommending the same diagnostic cut points established for the diagnosis of impaired glucose tolerance outside of pregnancy. In 1999, WHO clarified that GDM encompassed impaired glucose tolerance and diabetes (fasting ≥ 7 mmol/L, 2-h ≥ 7.8 mmol/L) and over the years has maintained their recommendations (5). The recently published Hyperglycemia and Adverse Pregnancy Outcomes (HAPO) study confirmed the link between hyperglycemia and adverse pregnancy outcomes, using the 2h 75-g OGTT with new cut points: FPG ≥ 5.1 mmol/L, 1-h ≥ 10.0 mmol/L, 2-h ≥ 8.5 mmol/L (6). In response to these results, the International Association of the Diabetes in Pregnancy Study Group (IADPSG) and American Diabetes Association (ADA) formulated new guidelines for screening and diagnosis of GDM (7).

There is no doubt from the literature that maternal glycaemia in women with GDM is involved in determining birth weight, but whether there is any influence of maternal glycaemia during OGTT on fetal over growth is unknown (8).

Black and cols. recently demonstrated that the risk of adverse pregnancy outcomes differs between women with impaired fasting plasma glucose values (FPG) and abnormal glucose levels during the OGTT, providing evidence that women with elevated FPG particularly suffer from delivering LGA infants (9). Ferrara and cols. found that both maternal FPG and 1-h, but not 2 or 3-h from 100-g. OGTT glucose levels were independent predictors of macrosomic risk (10). Caulfield and cols. evaluated risk of macrosomia according to abnormal glucose results and found that risk for infant macrosomia was higher among women with GDM only if they had fasting hyperglycemia (11). But, Kösüs and cols. found that 2 and 3 h glucose levels were the most important predictors of the macrosomia (12).

Excess nutrient delivery to the fetus causes LGA newborns, but whether fasting or peak glucose levels are more correlated with fetal overgrowth is less clear. So, we decided to explore the relation between the results of 2-h 75-g OGTT and neonatal birth weight.

The aim of this study was to investigate which glucose measurement from the 75-g OGTT (FPG, 1-h, and 2-h) has more capability of predicting large for-gestational-age (LGA) newborns of mothers with GDM.

## SUBJECTS AND METHODS

One hundred and eighteen pregnant women were prospectively screened for GDM between 24^th^ and 28^th^ week of pregnancy. The pregnant women were Outpatients of the Endocrinology, Diabetes, and Metabolic Disorders Department of the University Clinical Hospital in Skopje, R. Macedonia in a period from 01.2013 to 06.2013. GDM was defined according to the IADPSG (7), criteria for glucose measurements during 2-hours 75-g OGTT, as at least one level greater than fasting plasma glucose equal to or exceeding 5.1 mmol/L, 1 hour OGTT glucose equal to or exceeding 10.0 mmol/L, and 2 hour OGTT glucose equal to or exceeding 8.5 mmol/L. We followed participants until delivery and obtained delivery outcome data.

The following parameters were analyzed: patients’ ages, pre-pregnancy body mass index (BMI), BMI before delivery, FPG, 1 and 2 hour OGTT glucose values, haemoglobin A1c (HbA1c) at third trimester, gestational week of delivery, mode of delivery and baby birth weight.

All of the patients underwent 75-g OGTT, following the standard recommendations (13). Briefly, 75-g a hydrous glucose load was administered after a 12- to 14-h fast and fasting, 1-h post-load and 2-h post-load samples for glucose were obtained from an antecubital vein. Samples were collected in tubes containing fluoride and kept at 4°C until centrifugation up to 2 h later. Plasma measurements were performed with glucose oxidase methods.

The BMI of women was calculated by dividing the weights by the squared heights in meters (km^2^). The patients were weighed wearing clothes without shoes in the morning with an electronic scale at the first visit. Height was measured to the nearest 1 cm with a stadiometer. At enrollment pre-pregnancy BMI was calculated by using the most recent self reported weight before conception. Blood samples for HbA1c were taken after overnight fasting. HbA1c was measured by an ion-exchange HPLC instrument (DS5; Drew, USA) with a reference range of 4.2–6%. We followed participants until delivery. Neonatal outcomes were performed in University Clinic of Gynecology and Obstetrics. Mode of delivery was noted as spontaneous or caesarean section. Birth weight and proportion of LGA (defined as a birth weight > 90^th^ percentile for local population after adjusting for gestational age and sex) were determined. The gestational age of newborns was estimated from the date of the last menstrual period.

All patients gave informed consent to participate in the study after receiving explanations regarding the research protocol. The study was done according to the Helsinki Declaration.

### Statistical analyses

Statistical analyses were performed by SPSS 14.0 software. The significance of the differences between pregnant women with and without GDM was tested using t-test for numeric variables, and χ^2^-test for categorical variables. To determine the relationship between analyzed variables Spearman’s correlation test was used. In regression model all variables were grouped into one block and were entered in a single step using “enter” regression method. The variables with no collinearity (r < 0.70) were considered for further analyses. Coolinearity between the independent variables was assessed pair-wise by calculation of Spearman rang correlations. As goodness-of-fit statistics was used R square change if the R^2 ^change associated with a variable is large, that means that variable is a good predictor of the dependent variable. LGA was dependent variable. We plotted receiver operator characteristic (ROC) curves for predicting LGA newborns, and estimated and compared the area under the curve (AUC) using the MedCalc Statistical Software, version 12.5.0. Differences were considered significant if p was less than 0.05.

## RESULTS

From 118 included women, 78 (66.1%) were with GDM. The baseline data of all included pregnant women are presented in Table 1.

**Table 1:** Baseline characteristics of all included pregnant women

	GDM (n = 78)	Without GDM (n = 40)	p value
Age (years)	32.6 ± 4.8	31.4 ± 5.1	NS
Pre-pregnancy BMI (kg/m^2^)	26.4 ± 6.3	25.9 ± 4.4	NS
BMI before delivery (kg/m^2^)	**30.1 ± 4.9**	**26.5 ± 8.9**	**< 0.01**
HbA1c (%)	**5.2 ± 1.2**	**4.7 ± 0.2**	**< 0.01**
FPG (mmol/L)	**5.8 ± 1.2**	**4.7 ± 0.5**	**< 0.01**
1-hour OGTT (mmol/L)	**11.3 ± 1.8**	**7.4 ± 1.4**	**< 0.01**
2-hour OGTT (mmol/L)	**9.3 ± 2.1**	**6.2 ± 1.2**	**< 0.01**
Birth weight (g.)	**3,569 ± 681**	**3,267 ± 501**	**< 0.05**
LGA (%)	**24/78 (30.7)**	**2/40 (5.0)**	**< 0.01**
Cesarean section (%)	37/78 (47.4%)	9/40 (22.5%)	NS
Gestational week of delivery	38.8 ± 1.8	39.3 ± 1.3	NS

Displayed results are average ± std deviation and percentages. Comparisons between the 2 groups were performed by student’s t-test for continuous variables and χ^2 ^–test for categorical variables.

BMI: body mass index; HbA1c: glycosylated haemoglobin; FPG: fasting plasma glucose; OGTT: oral glucose tolerance test; LGA: large for gestational age; NS: no significance.

As expected, women with GDM were older, more overweight before pregnancy, and with higher weight gain than women with normal glucose tolerance, but the differences were slight, so they did not reach statistical significance. There were statistically significant differences between proportions of LGA newborns from GDM and normal pregnancies. The incidence of cesarean section was not different between the women with GDM and women without GDM (Table 1).

There were significant correlations between LGA newborns from GDM pregnancies with BMI before pregnancy (r = 0.36, p < 0.01), BMI before delivery (r = 0.36, p < 0.01), FPG (r = 0.46, p < 0.05), and 1-h OGTT plasma glucose levels (r = 0.30, p < 0.05). So, r value from correlation analyses for variable FPG was greater than other variables: pre-pregnancy BMI, BMI before delivery and 1-h OGTT. We also noted significant correlations between FPG with BMI before pregnancy (r = 0.47, p < 0.01), BMI before delivery (r = 0.47, p < 0.01), and HbA1c (r = 0.25, p < 0.05).

The results from multiple regression analysis which determine independent risk factors for LGA are shown in Table 2. Gestation week of delivery and FPG were independent predictors for LGA (Beta = 0.58 and Beta = 0.37 respectively, p < 0.01) (Table 2).

**Table 2:** Influence of maternal characteristics on LGA newborns from GDM pregnancies

	Unstandardized coefficients		Standardized coefficients	t	Sig.
	B	Std. Error	Beta		
(Constant)	-0.751				
pre-preg BMI	.266	.172	.193	1,543	.128
BMI before delivery	.779	.240	.004	0.324	.747
HbA1c	.778	.595	.016	1,307	.196
FPG	**.206**	**.649**	**.377**	**3,185**	**.002**
1h-OGTT	.146	.403	.004	.36	.717
g.w. of delivery	**.216**	**.037**	**.598**	**5,834**	**< .0001**

Multiple regression analysis model with large for gestational age as a dependent variable, according to maternal characteristics from pregnancies of women with gestational diabetes was used.

BMI: body mass index; HbA1c: glycosylated haemoglobin; FPG: fasting plasma glucose; OGTT: oral glucose tolerance test; g.w.: gestational week.

**Figure 1:**
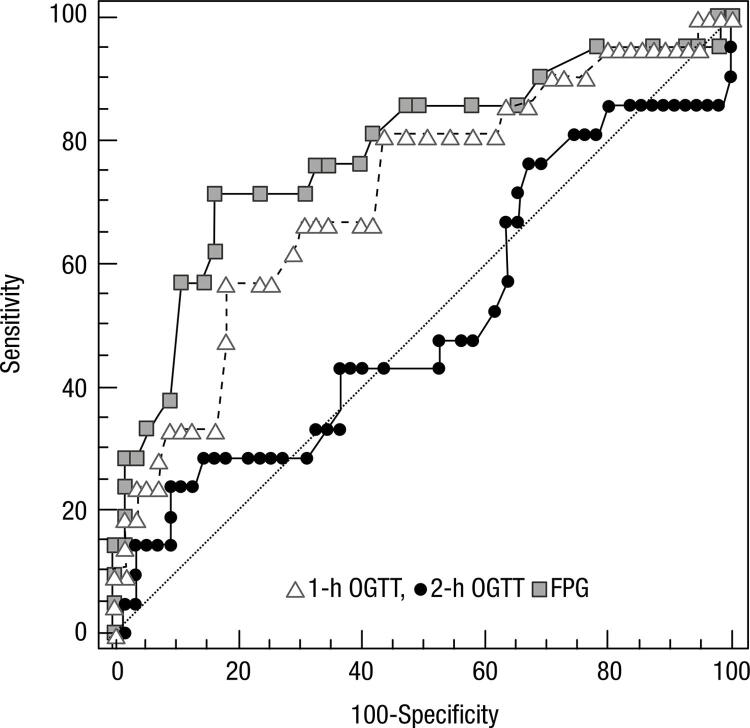
Receiver operator characteristics curves comparing performance of fasting, 1-h and 2-h OGTT glucose levels for the prediction of LGA. The true positive rates (sensitivity) versus the false positive rates (100-specificity) are plotted for the cut off glucose levels (each data point is 0.2mmol/L apart).

In order to evaluate fasting, 1-h and 2-h OGTT glucose levels for the prediction of LGA, we compared the areas under the receiver operator characteristic curve (AUC) of the ROC curves. Figure 1 shows that, for LGA newborns from GDM pregnancies, fasting AUC was 0.782 (0.685-0.861), for 1-h OGTT was 0.719 (0.607-0.815), and for 2-h OGTT was 0.51 (0.392-0.626). Fasting glucose levels and 1-h OGTT glucose levels show statistically significant predictability for LGA (p < 0.05). Thus, FPG shows higher predictability than 1-h OGTT glucose levels.

OGTT: oral glucose tolerance test, FPG: fasting plasma glucose.

## DISCUSSION

The results of the study confirmed that gestational diabetes pregnancies were complicated with statistically higher percent of LGA newborns than normal pregnancies. We also showed that values at 1-hour from 75-g OGTT have the strongest association with LGA newborns from women with GDM pregnancies.

Disproportion in the number of patients with GDM and normal pregnancies in this study is due to the implementation of the study in tertiary health institution, where obstetricians sent pregnant women with more risk factors for GDM.

It is known that fetal overgrowth is driven by a cluster of metabolic alterations associated with maternal obesity. Birth weight is largely determined by maternal factors such as prepregnancy BMI, weight gain during pregnancy and maternal serum lipid levels in mothers with GDM (14-16). We did not analyze lipid parameters, but pre-pregnancy BMI and BMI before delivery significantly correlated with LGA. In their study Charlotte and cols. concluded that maternal obesity had the strongest and independent effect on LGA in pregnancies complicated by GDM (17). According to them, incidence of LGA infants was significantly higher in obese women than in those with normal BMI, which is comparable with our findings. However, in our study we found statistically significant differences only in BMI before delivery. It is well known that FPG and OGTT glucose levels are mirror for metabolic alterations in women with GDM. FPG and 1-h OGTT glucose levels correlated and were independent risk factors for LGA. FPG will increase the risk for LGA newborns in women with GDM pregnancies especially when it is combined with higher BMI before delivery. In our study regression analysis did not show that pre-pregnancy BMI and BMI before delivery are independent predictors for LGA. This may be explained by the fact that our patients were overweight, but not obese or severely obese. Pre-pregnancy BMI and BMI before delivery were 26 ± 6.3 and 30.1 ± 4.9 kg/m^2^, respectively.

Also, this study did not find a correlation between HbA1c and LGA, nor was HbA1c an independent predictor for LGA. Other studies reported that the relationship between HbA1c and neonatal birth weight is weak (18,19). It is well known that HbA1c is not an appropriate measure for the prediction for LGA babies. There is a large overlap in the distribution of HbA1c values between women with normal, borderline abnormal and mildly abnormal blood glucose values (20). Therefore, HbA1c is not a suitable test to detect mildly impaired glucose tolerance. The mean HbA1c in this study was 5.2 ± 1.2%.

Fasting plasma glucose levels and 1-h OGTT glucose levels were more powerful in prediction of LGA than 2-h OGTT glucose levels in this study. Identical with our results, Ouzilleau and cols. found that FPG levels correlated better with birth weight than the 2-h levels from 75-g OGTT in univariate, multivariate analyses, and ROC curves (21). Mello and cols. show that glucose levels at 1-h from 75-g OGTT gave a strong association with all abnormal neonatal anthropometric characteristics including LGA (22). Perucchini and cols. recommended universal screening for GDM using a FPG of ≥ 4.8 mmol/L between 24th and 28^th^ gestational week as an easier screening procedure (23). Fasting plasma glucose measurement is well tolerated and an inexpensive routine examination. So, FPG is a simple indicator for identifying high risk women with GDM. Earlier reports noted that the fasting glucose value was better than the postchallenge glucose levels in identifying LGA risk (21,24,25). Similarly, Retnakaran and cols. found that FPG best predicts LGA risk, whereas post load glucose levels from OGTT predict postpartum prediabetes or diabetes risks (26). When we identify the strongly associated level of OGTT with the risk of LGA newborn, the treatment strategy for clinicians can be easy.

The investigators from HAPO study found a continuum of increasing risk of adverse outcome as each of the three (FPG, 1-h, and 2-h) plasma glucose values increased (6). These adverse outcomes included LGA, cesarean delivery, neonatal hypoglycemia, and preeclampsia. In conclusion, if more than one level is exceeded, the chances for delivering LGA babies are higher. According to our results, the combination of higher FPG and 1-h values from the 75-g OGTT gives higher predictability for LGA. So, tight glucose control in these target women may be necessary to avoid LGA newborns.

The weakness of the study is the small cluster of women and the influence of the GDM treatment on birth weight. All women were treated according to their glycemic profile with tendency to achieve fasting, postprandial and HbA1c values recommended for pregnant women with GDM. In spite of treatment 30% of newborns were LGA. The question remains whether we need to take into account FPG values to determine the optimal time for starting with therapy and the kind of therapy, diets or insulin. Large controlled interventional clinical trials should be done to answer this question.

In conclusion our study documented a significant association between fasting and 1-h 75-g OGTT glucose levels with LGA newborns.
